# Analysis of the anti-proliferative and the pro-apoptotic efficacy of Syk inhibition in multiple myeloma

**DOI:** 10.1186/s40164-015-0016-z

**Published:** 2015-08-05

**Authors:** Ruth-Miriam Koerber, Stefanie Andrea Erika Held, Annkristin Heine, Philipp Kotthoff, Solveig Nora Daecke, Anita Bringmann, Peter Brossart

**Affiliations:** Medical Clinic III, Department of Hematology and Oncology, University Hospital Bonn, Sigmund-Freud-Str. 25, 53127 Bonn, Germany

**Keywords:** Spleen tyrosine kinase (Syk), Syk inhibitors, Multiple myeloma, Plasmacell malignancy, Tyrosine kinase, Apoptosis, Signal transduction Bay61-3606, Piceatannol, R406

## Abstract

**Background:**

Multiple myeloma (MM) is a clonal B cell malignancy characterized by proliferation of malignant plasma cells in the bone marrow. Despite high-dose melphalan therapy with autologous stem cell transplantation (ASCT) and the introduction of immunomodulatory drugs like bortezomib or lenalidomide, that have been associated with improved survival, MM is still incurable and new treatment options are needed. In B cell malignancies such as chronic lymphocytic leukaemia (CLL) or diffuse large B cell lymphoma (DLBCL), Syk (spleen tyrosine kinase) inhibitors have shown promising in vitro and first clinical results. In our study, we analyzed the potential of Syk as a target in MM.

**Methods:**

The MM cell lines AMO-1, U266 and RPMI8226 and primary MM cells were treated with the Syk inhibitors BAY61-3606, R406 or Piceatannol and proliferation, migration and apoptosis induction were analyzed. Effects on involved intracellular signaling cascades were determined by Western blotting. Furthermore, we analyzed synergistic and additive effects of Syk inhibitors in combination with established anti-myeloma drugs and experimental inhibitors (e.g. PI-3-Kinase inhibitor NVP-BEZ235).

**Results:**

Incubation of MM cell lines as well as primary MM cells with Syk inhibitors resulted in a reduced proliferation and stromal cell-derived factor-1 alpha (SDF-1 alpha) induced migration that was accompanied by a concentration dependent inhibition of the MAP-Kinase, characterized by reduced phosphorylation of ERK an p38 molecules, and NF-kappaB signalling pathways. Furthermore, Syk inhibition induced apoptosis in MM cells in a dose-dependent manner, characterized by reduced expression of pro-caspase 3, increased PARP-1 cleavage and enhanced release of cytochrome *c*. In addition combined treatment of MM cells with Syk inhibitors and NVP-BEZ235 (dual PI3-kinase/mTOR inhibitor) or MAPK inhibitors (PD98059, SP600125, U0126, SB203580) resulted in increased apoptotic activity of the drugs.

**Conclusions:**

Our results show that Syk inhibition might represent a promising new treatment option in MM with an increased efficacy when combined with MAP kinase inhibitors. Furthermore, our study strongly underlines the potency of Syk inhibitors as a potential therapeutic treatment option for MM patients.

**Electronic supplementary material:**

The online version of this article (doi:10.1186/s40164-015-0016-z) contains supplementary material, which is available to authorized users.

## Background

Multiple myeloma (MM) is a B cell clonal malignancy characterized by proliferation and accumulation of malignant plasma cells within the bone marrow (BM) [1]. Several recently introduced therapies such as bortezomib, thalidomide or lenalidomide as well as high dose chemotherapy and autologous stem cell transplantation (ASCT) have improved the prognosis and overall survival of patients with MM. However, MM is still an incurable disease [2, 3].

Drug resistance represents one of the major difficulties to overcome in patients with malignant disease which often results in failure to eliminate minimal residual tumor cells [4, 5]. Studies performed in an attempt to determine the factors mediating drug resistance and survival of malignant cells led to the characterization of novel pathways and identification of potential targets for anticancer therapies [6, 7]. In malignant B cells and plasma cells, the involvement of signalling pathways via Janus kinases 1/2 (JAK1/2) and the signal transducers and activators of transcriptions (STAT) molecules as well as the events mediated by the phosphatidylinositol-3 kinases (PI3-K) and mitogen activated kinases (MAPK) were found to be critical for cell growth, differentiation and survival [8–10].

In addition, emerging evidence suggests that spleen tyrosine kinase (Syk), an intracellular tyrosine kinase, plays a central role upon activation of cells by B or T cell receptors (BCR or TCR) as well as by cytokines or adhesion molecules [11–13]. Syk activation is mediated when sarcoma kinases (Src kinases) phosphorylate conserved sequences within receptors containing immune tyrosine activation motifs (ITAMs) [14, 15]. Once activated, Syk propagates the phosphorylation of further downstream molecules including the PI3-K and MAP-kinase pathways [16, 17].

Syk and the zeta-chain-associated protein kinase 70 (ZAP-70) are the only members of the Syk family kinases. Syk was first identified by isolation of tyrosine kinase activity found in the thymus and spleen leading to the cloning of the 72 kDa protein [18, 19]. Mice with genetically disrupted Syk are deficient in B cell maturation and expansion and die prenatally due to severe bleedings. This indicates that Syk might play a role in angiogenesis and maintaining of vascular integrity [20, 21]. Subsequently, Syk has been found to be involved and play a broad role in hematopoietic cell signalling such as FCeRI receptor in mast cells, FCgRIIA in macrophages and platelets as well as the antigen receptors of B and T cells [22, 23]. In addition, it was demonstrated that Syk is also expressed in fibroblasts, hepatocytes, neuronal cells and vascular endothelium [24]. Syk was shown to function as an oncogene and to be a negative regulator of tumorigenesis in some malignant diseases such as breast cancer, B and T cell lymphoma and myelodysplastic syndromes [25, 26].

Recently, several reports on preclinical studies demonstrated that Syk inhibition might represent a rational therapeutic target in B cell malignancies such as chronic lymphocytic leukaemia (CLL), diffuse large B-non-Hodgkin lymphoma (DLBCL), follicular and mantle cell lymphoma by interfering with the signalling promoted by the activation via the BCR, cytokines and stromal factors [27–29]. In DLBCL and CLL, first clinical experiences with the clinically available Syk inhibitor R406 provided encouraging and promising results in heavily pre-treated patients [30–32].

In our study we analyzed the effects of the three currently available Syk inhibitors, Piceatannol, Bay-61-3606 and R406 on the viability and functional properties of myeloma cells in order to provide a mechanistic understanding and a rationale for a possible use of these compounds as therapeutic option.

## Results

### Syk inhibitors reduce the proliferation and migration of MM cells

**Fig. 1:**
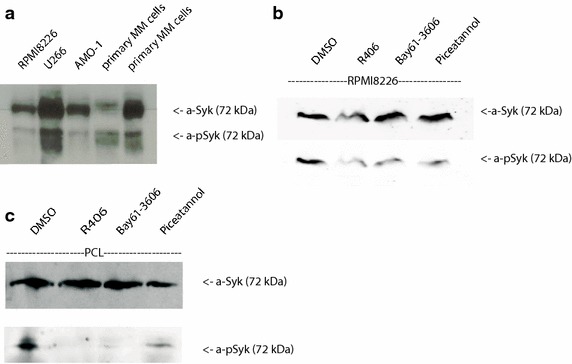
**a**, **b** Expression of Syk and phosphorylated Syk in different cell lines and patient samples. **a** Syk was immunoprecipitated from MM cell lines and primary MM cells. Immunoprecipitates were subjected to Western blotting using specific antibodies. Primary myeloma cells were obtained from peripheral blood of myeloma patients with plasma cell leukemia (PCL, purity >90%). Displayed is a representative immunoblot of Syk and pSyk in the absence of Syk inhibitors which shows Syk expression in the utilized MM cell lines and PCL-cells. **b** and **c** Addition of Syk inhibitors (Piceatannol, R406 and Bay61-3606) reduce the expression of pSyk in MM cell lines and PCL cells. Ponceau S staining was performed to confirm equal amounts of protein.

Syk was shown to play an important role in the signal transduction via the BCR, cytokine receptors, integrins and Fc receptors on normal and malignant B cells. These receptors promote survival and are associated with the induction of proliferation, differentiation and migration. Using Immunoprecipitation, we found that phosphorylated Syk is expressed in the utilized multiple myeloma cell lines AMO-1, U266 and RPMI8226. Furthermore, Syk expression was also detected in primary MM cells of patients with plasma cell leukemia (PCL). In addition, we found that Syk can be downregulated when these myeloma cells were incubated with the inhibitors of Syk phosphorylation, i.e. Bay 61-3606 or Piceatannol and R406 (Fig. 1a, b).

**Fig. 2:**
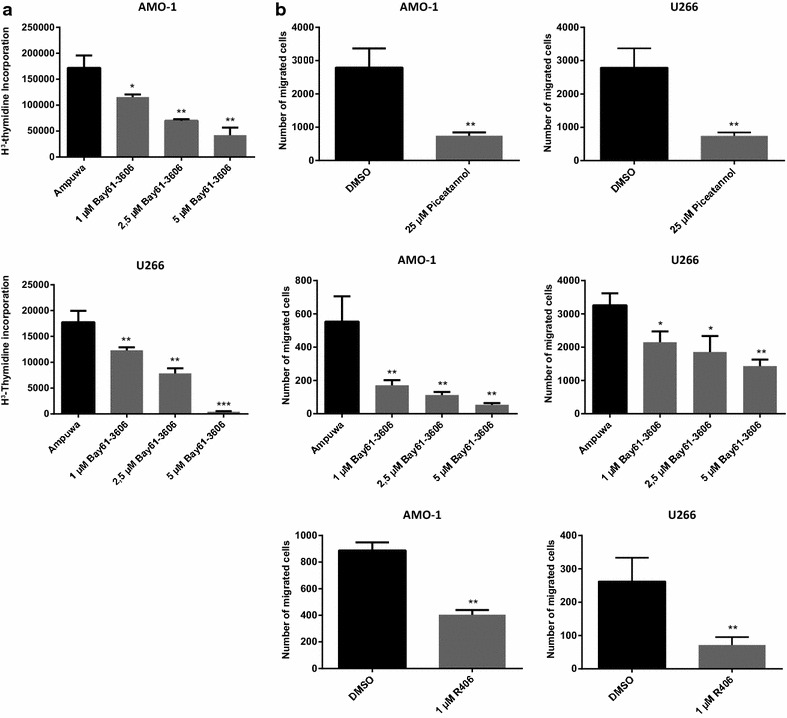
**a**, **b** Syk inhibitors reduce proliferation and migration in a dose-dependent manner. **a** MM cell lines were incubated with Syk inhibitors (Piceatannol or Bay61-3606) for 24 h and the proliferative response was analyzed by ^3^H-thymidine uptake. Syk inhibition resulted in a decrease of cell proliferation. **b** Migration of MM cells towards SDF-1α were performed with or without Syk inhibitors (Piceatannol, Bay61-3606 and R406) using transwell chemotaxis assays. The percentage of migrated cells was determined after 3 h by flow cytometry. Syk inhibitors diminished the migration towards SDF-1α stimulated cells. The shown results are representative for all tested MM cell lines. Results are expressed as the mean of three panels per pattern. Results are shown from one experiment representative of at least three. DMSO or Ampuwa was used as control. The significance is related to DMSO/Ampuwa. ^+^P < 1.000, *P < 0.1, **P < 0.01, ***P < 0.001.

Incubation of MM cell lines with Piceatannol, R406 or Bay 61-3606 led to a concentration dependent decrease of proliferation, as analyzed by ^3^H-thymidine incorporation, and to a reduced stromal cell-derived factor-1 α (SDF-1α) induced migration of cells in transwell experiments as depicted in Fig. 2a and b for the AMO-1 cells. Similar results were obtained with other MM cell lines (U266 and RPMI8226).

Additionally, we performed MTT (tetrazolium salt 3-4,5-dimethylthiazol-2yl]-2,5-diphenyl-tetrazolium bromide) cytoxicity assays to ensure that the effects on migration and proliferation induced by Syk inhibitors are not due to cytotoxicity as a mechanism of action. The MTT assays showed no decline in cell viability when incubated with Syk inhibitors. This was proved in three cell lines at three different concentrations. Based on these results, the effects of the various Syk inhibitors on migration and proliferation are not due to cytotoxic effects (see Additional file 1).

**Fig. 3:**
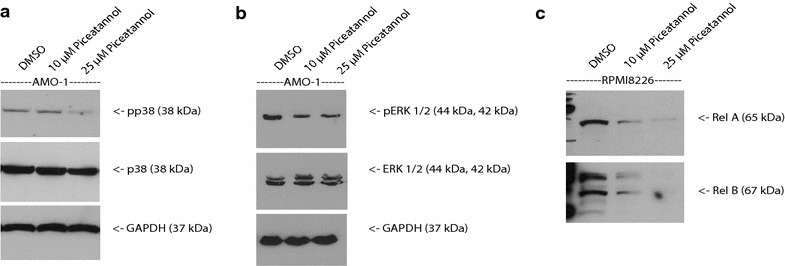
**a**–**c** Syk inhibition reduces phosphorylation of p38 and ERK1/2 in MM cell lines. **a**, **b** MM cell lines were treated with Piceatannol at concentrations of 10 and 25 µM. Phosphorylation status of down stream signaling mediators p38-Kinase and ERK1/2-Kinase (MAPK p42 and p44) was evaluated by Western blotting using antiphospho-p38 and antiphospho-ERK 1/2 antibodies. GAPDH was used to confirm equal amounts of protein. Results are representative of at least three independent experiments. **c** Nuclear translocation of NF-κB family members was determined by Western Blot analysis of nuclear extracts upon incubation with Syk inhibitors (**c**). Piceatannol was applied at 10 and 25 µM. DMSO was used as control. Proteins detected are indicated by an *arrow* on the *right*. Ponceau S staining was performed to confirm equal amounts of protein. Results are representative of at least three independent experiments.

In line with these results, we found that treatment of cells with the compounds resulted in a reduced activation of the downstream signalling events characterized by reduced phosphorylation of extracellular signal-regulated kinases 1/2 (ERK1/2) and p38 mitogen-activated protein kinases (Fig. 3a, b) and nuclear localized expression of transcription factors of the nuclear factor kappa-light-chain-enhancer of activated B cells (NF-kB) family RelB and RelA (Fig. 3c). Representatively, the results are shown for AMO-1 cell line. However, all used MM cell lines (U266 and RPMI-8226) showed similar results in our experiments. The utilized concentrations of Syk inhibitors were adjusted to reported working concentrations in the literature.

## Inhibition of Syk induces apoptosis in MM cells

**Fig. 4:**
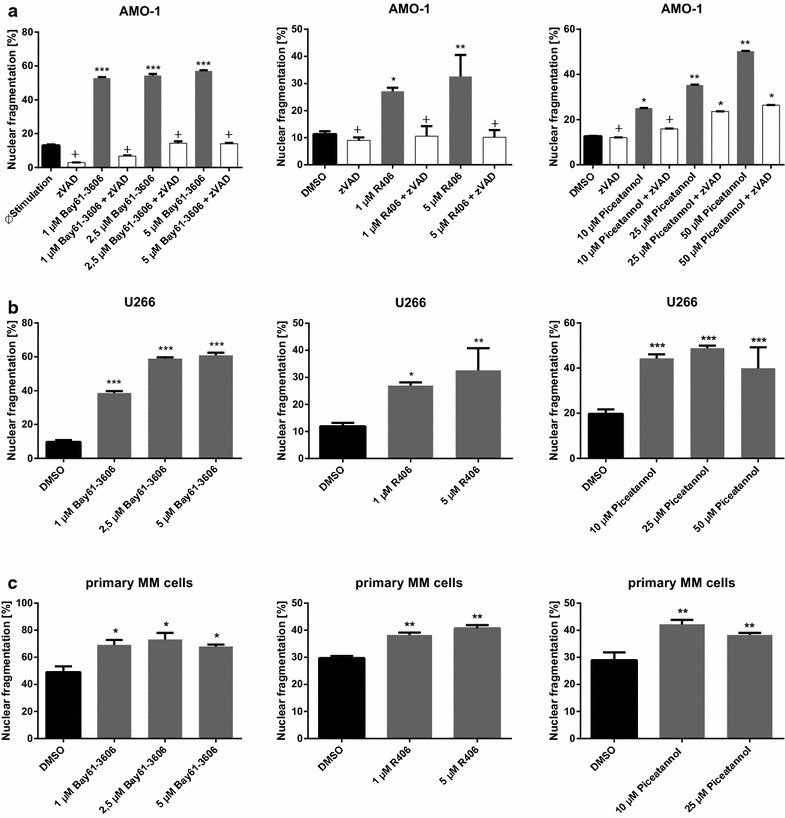
**a**–**c** Inhibition of Syk induces apoptosis in MM cell lines. **a**–**c** Viability of MM cells was defined by the amount of nuclear fragmentation and compared to the respective DMSO control. MM cell lines [AMO-1 (**a**), U226 (**b**) and primary MM cells from patients with plasma cell leukemia (purity >90%) (**c**)] were treated with various concentrations of Syk inhibitors (Piceatannol, R406 and Bay61-3606) as indicated. After 24 h of incubation with Syk inhibitors, propidium iodide-staining was performed. Nuclear fragmentation was determined by flow cytometry. zVAD-FMK was added as confirmation for caspase activation (*white bar graphs*). **c** Primary MM cells were isolated from peripheral blood from patients with plasma cell leukemia (purity >90%) and analyzed as described above. Results are expressed as the mean of three panels per pattern. Results are from one experiment representative of at least three. The significance is related to DMSO as control. ^+^P<1.000, *P < 0.1, **P < 0.01, ***P < 0.001.

In order to analyze the effect of Syk inhibitors on the viability of established MM cell lines, Piceatannol, R406 or Bay61-3606 were added to the cell culture medium and flow cytometric propidium iodide (PI) analysis of DNA fragmentation was performed as described previously [33]. Incubation of cells with the compounds resulted in a concentration dependent cell death induction that was inhibited by the addition of the pan-caspase inhibitor zVAD-FMK. This indicates that caspase activity was indispensable for the observed apoptotic effects (Fig. 4a, b).

Likewise, we wanted to determine the efficacy of the compounds to induce apoptosis in purified MM cells. As shown in Fig. 4c, incubation of primary MM cells from patients with plasma cell leukemia with R406, Piceatannol or Bay61-3606 significantly reduced cell viability.

**Fig. 5:**
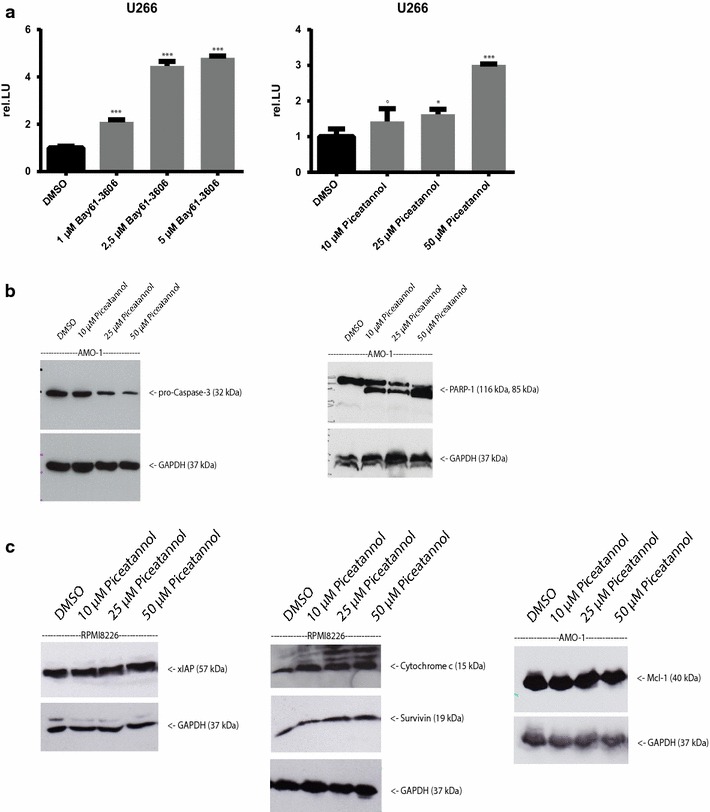
**a**, **b** Caspase-3 activity is increased significantly upon treatment with Syk inhibitors. **a** Caspase-3 activity was determined from whole cell lysates by the cleavage of the fluorogenic caspase substrate *N*-acetyl-Asp-Glu-Val-Asp-aminomethylcoumarin (DEVD-AMC). The release of aminomethylcoumarin was determined by fluorometry using an excitation length of 360 nm and an emission wave-length of 460 nm. Syk-Inhibitors induce a significant concentration-dependent increase of caspase-3 activity. Results are expressed as the mean of three panels per pattern. Results are from one experiment representative of three. DMSO was used as control. The significance is related to DMSO. ^+^P<1.000, *P < 0.1, **P < 0.01, ***P < 0.001. **b** Western Blot analyses of AMO-1 whole cell lysates treated with Piceatannol was utilized to analyze the expression of pro-caspase 3 and PARP-1 cleavage. After 24 h of incubation with Syk inhibitors, whole cell lysates were prepared and Western Blot analysis was performed. DMSO was used as a control. GAPDH was used to confirm equal amounts of protein. Results are representative of at least three independent experiments. Proteins detected are indicated by an *arrow* on the *right.* **c** Expression of xIAP, Mcl-1, cytochrome *c* and Survivin upon treatment with Syk inhibitors. Protein extracts were analyzed for the expression of proteins participating in apoptosis such as xIAP, Mcl-1, survivin and cytochrome *c* by immunoblotting. An increase of cytochrome *c* was observed, implicating that Piceatannol induces apoptosis via the internal, mitochondrial pathway. There was no effect of Piceatannol on the anti-apoptotic proteins survivin, Mcl-1 and xIAP. Results are representative of various independent experiments. Proteins detected are indicated by an *arrow* on the *right*.

Caspase-3 assays and Western Blot analysis revealed that the induction of apoptotic cell death was accompanied by caspase-3 activation and poly[ADP ribose]polymerase 1 (PARP-1) cleavage (Fig. 5a, b). In addition, an increased release of cytochrome *c* upon incubation with the compounds indicated that the apoptosis induction in tested MM cells was mediated via the mitochondrial signalling pathway (Fig. 5c). In contrast to previous findings in CLL cells, we could not detect any regulation of the expression of myeloid leukaemia cell differentiation protein MCL-1, the x-linked inhibitor of apoptosis protein xIAP or survivin (also known as BIRC5 or API4) by the used compounds (Fig. 5c).

**Fig. 6:**
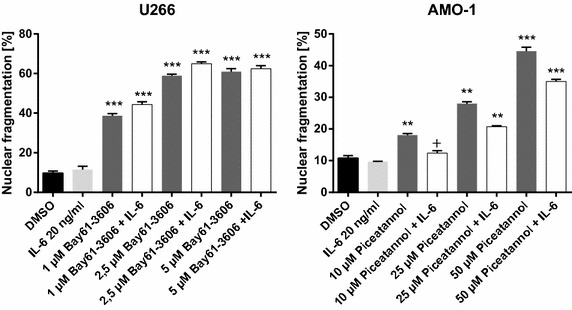
Efficiacy of Syk inhibition on cell viability is not reduced in the presence of Il-6. To determine the impact of IL-6 on the apoptotic effects of Syk inhibitors, MM cells were preincubated with IL-6 and nuclear fragmentation of MM cells treated with Syk inhibitors was measured by flow cytometry. In comparison with Bay61-3606 or Piceatannol alone, a combination with IL-6 did not reduce significantly the number of apoptotic cells. Results are expressed as the mean of three panels per pattern. Results are from one experiment representative of three. DMSO was used as control. The significance is related to DMSO. ^+^P<1.000, *P < 0.1, **P < 0.01, ***P < 0.001.

IL-6 is a major growth and drug-resistance factor for MM cells that activates a cascade of signalling pathways mediating proliferation and anti-apoptotic effects. We therefore analyzed whether IL-6 might interfere with and abrogate the apoptotic effects induced by Syk inhibitors. Preincubation of MM cells with IL-6 had no effect on Piceatannol triggered apoptosis in MM cell lines (Fig. 6). Similarly, preincubation of MM cell lines with TLR ligands (TLR 2, 3, 4, 7/8) had no effect on the action of Syk inhibition (data not shown).

Because bone marrow (BM) microenvironment promotes MM survival and drug resistance, we next determined the apoptosis induction in MM cells in this context. The majority of MM patients expresses the chemokine receptor CXCR4 on neoplastic MM cells which is crucial for myeloma cell migration and homing to the BM microenvironment. Additionally, it supports tumor cell survival and mediates chemotherapy resistance. For contacts of cell–cell and cell to extracellular matrix, a group of cellular adhesion molecules such as CD11a or CD49d are essential, likewise their ligands, i.e. CD102 (ICAM 2). Surface antigens were examined by flow cytometric analysis. Despite reduced migration of cells when treated with Syk inhibitors, we could not detect any effect on the expression of CXCR4, CD49d or CD102 on the cell surface of MM cells (data not shown).

## Combined treatment of MM cells with Syk inhibitors

**Fig. 7:**
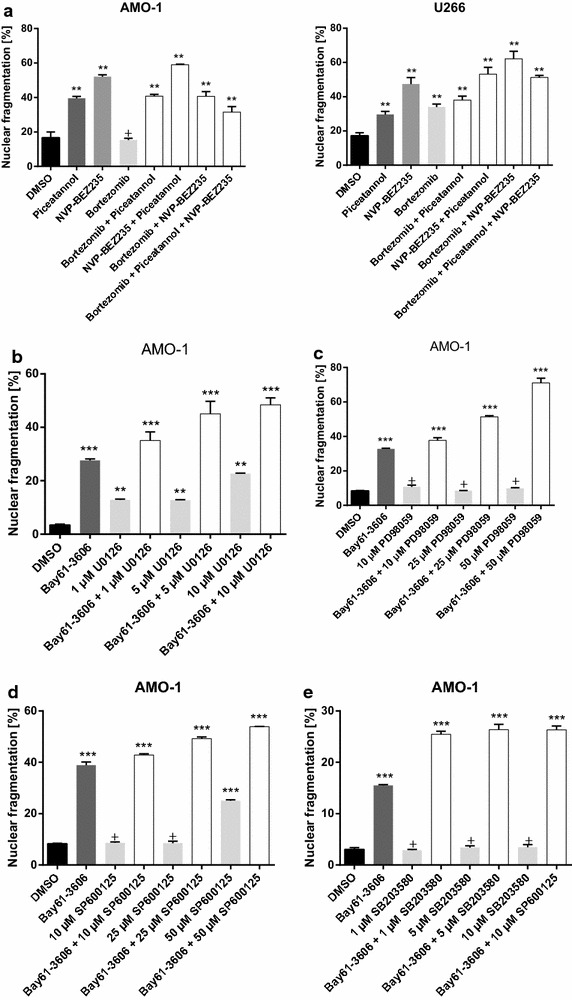
Combined treatment with NVP-BEZ235 or MAPK inhibitors increases induction of apoptosis. **a** Nuclear fragmentation of MM cells treated with either/and 500 nM NVP-BEZ235, 25 µM Piceatannol and 0.5 ng bortezomib. Nuclear fragmentation was determined by the method of Nicoletti and analysed by flow cytometry. In comparison to Piceatannol alone, a combination with the common anti-myeloma agent bortezomib exerts no further increase of nuclear fragmentation. The addition of NVP-BEZ235 (an orally bioavailable dual phosphatidylinositol 3-kinase/mammalian target of rapamycin inhibitor) to Syk inhibitors increased nuclear fragmentation. Statistical analysis showed, that the combination of NVP-BEZ235 and Piceatannol is significantly higher than each drug alone. This works predominantly for AMO-1 cells. Calculational significant results in U266 cells can only be observed when correlated to Piceatannol, but not to NVP-BEZ235 (see Additional file 1). Results are expressed as the mean of three panels per pattern. Results are from one experiment representative of three. DMSO was used as control. The significance is related to DMSO. ^+^P<1.000, *P < 0.1, **P < 0.01, ***P < 0.001. **b**–**e** Effects of Syk inhibition in combination with MAP-Kinase inhibitors are demonstrated. MM cell lines were incubated with 5 µM Bay61-3606 in addition to varying concentrations of U0126 (**b**), SP600125 (**c**), PD98059 (**d**) or SB202190 (**e**) alone as well as in combination. Combined treatment of Bay61-3606 with U0126 (a potent, selective inhibitor of MAP2K), SP600125 (a selective inhibitor of JNK), or PD98059 (an inhibitor of MAP2K/MEK) and SB203580 (a selective inhibitor of p38 MAPK) results in significant synergistic or additive effects. Results are expressed as the mean of three panels per pattern. Results are from one experiment representative of three. DMSO was used for negative control. The significance is related to DMSO. ^+^P<1.000, *P < 0.1, **P < 0.01, ***P < 0.001.

In the next set of experiments we analyzed possible additive effects of drugs currently used in the treatment of MM with Syk inhibitors. We found no additive effects when Syk inhibitors were used with bortezomib (Fig. 7a). In addition, no additive effects were detected when Syk inhibitors were combined with thalidomide, lenalidomide, TRAIL (tumor necrosis factor related apoptosis inducing ligand) or dexamethasone (data not shown).

NVP-BEZ235, an orally available dual PI3 kinase/mTor inhibitor, was recently shown to suppress survival and growth of MM cells in preclinical studies. We found that combined treatment of MM cells with Piceatannol and NVP-BEZ235 significantly increased the cytotoxic effect as compared to each drug alone (Fig. 7a). This was predominantly observed in AMO-1 cells. Significant results could be observed in both cell lines when correlated to Piceatannol, in U266 cells significances correlated to NVP-BEZ235 were not significant, but in AMO-1 they were significantly higher (see Additional file 1).

As mentioned above, MAP-Kinases play a critical role in differentiation, cell growth and survival in MM. Combination of Syk inhibitors with MAP-Kinase inhibitors such as U0126 (a potent, selective inhibitor of MAP2K), PD98059 (an inhibitor of MAP2K/MEK), SP600125 (a selective inhibitor of JNK) and SB203580 (a selective inhibitor of p38 MAPK) resulted in synergistic cytotoxic effects in combination with U0126 (Fig. 7b) and PD98059 (Fig. 7d). Additive effects were observed when combined with SP600125 (a selective inhibitor of JNK, Fig. 7c) or SB203580 (Fig. 7e).

## Discussion

In our study we demonstrate that a proportion of MM cells express Syk and pSyk and Syk inhibitors R406, Piceatannol and Bay61-3606 effectively block proliferation, migration and survival of MM cells. This indicates that Syk inhibition might represent a potential new therapeutic option in the treatment of MM. However, the compounds we used in our experiments are not entirely specific for Syk and do display some lower activity against JAK, Lck, Lyn, STAT3 and STAT 5 as well as against some potential additional, yet to be determined, tyrosine kinases [34, 36, 37].

R406 and BAY61-3606 have previously been reported to inhibit proliferation and to induce apoptosis in non-Hodgkin lymphoma (B-NHL) and CLL cells [27, 38]. R406 is currently used in clinical trials and has demonstrated promising activity in patients with B-NHL, ITP and rheumatoid arthritis [39, 40]. The so far reported side effects of the compound are generally mild and consist primarily of gastrointestinal side effects and neutropenia, which was dose related and rapidly reversible upon dose reduction or discontinuation of treatment [41]. Interestingly, in mice treated with R406, additional immunomodulatory effects including lymphopenia, reduced thymus and spleen weight and decreased cellularity of the BM were observed, which resolved within 2 weeks after stopping the drug application [42].

MM cells express the chemokine receptor CXCR4 and its ligand SDF-1α controls their α4β1-mediated adhesion to the vascular adhesion molecule 1 (VCAM-1), fibronectin and endothelial cells as well as their transendothelial migration and homing in the BM [43, 44]. MM cells are highly dependent on BM microenvironment, where signals provided by cytokines and adhesion molecules promote the survival, proliferation and expansion of MM cells [45, 46]. In our study we found that treatment of MM cells with Syk inhibitors blocked the SDF-1α mediated migration of MM cells and abrogated the survival signals provided by IL-6, TLR ligands or BM stroma. These blocking effects were accompanied by the inhibition of downstream pathways as demonstrated by reduced ERK1/2 and p38 phosphorylation and nuclear localized RelB and RelA, indicating that MAP-Kinase and NF-κB signalling pathways are involved in the observed inhibitory effects.

Syk inhibitors efficiently induced apoptosis in established MM cell lines and purified MM cells. This was characterized by an enhanced caspase-3 activity and PARP-1 cleavage, a known caspase-3 substrate. The induction of cell death was inhibited by the addition of the pan-caspase inhibitor zVAD-FMK, further confirming the critical role of caspases in this setting. In addition, we found that Syk inhibition resulted in enhanced release of cytochrome *c* indicating that the apoptotic cell death was mediated via the internal, mitochondrial pathway.

Our findings are in line with previous findings in CLL cells [38] where treatment of CLL cells with BAY61-3606 caused caspase-3 activation, cleavage of PARP-1 and loss of mitochondrial potential. However, in contrast to this report we could not detect any regulation of MCL-1 protein expression in our experiments indicating that the effects and mechanisms induced by the utilized compounds may vary depending on the used cell lines and models.

The introduction of recently developed agents such as the proteasome inhibitor bortezomib or lenalidomide has dramatically improved the prognosis and overall survival in MM patients [47, 48]. Some of the induced effects by these targeted therapies are mediated by interfering with the MAP-Kinase and NF-kB signalling pathways. Therefore, we hypothesized that Syk inhibition might represent a rationale combination partner. While there were no additive effects by bortezomib, the combined treatment of MM cells with MAP-Kinase inhibitors resulted in an increased cytotoxic effect. In addition, we observed that simultaneous exposure to NVP-BEZ235 [49], an orally available dual inhibitor of PI3 kinase/mTor signalling significantly enhanced the efficacy of Syk inhibitors.

## Conclusions

Syk inhibitors already showed promising results in B cell malignancies such as CLL and DLBCL. Our data show successful findings of Syk inhibition in MM. Syk inhibition in MM resulted in decreased proliferation and migration of MM cells. Additionally, Syk inhibition induces apoptosis and is effective in combination with established anti myeloma drugs and experimental new kinase inhibitors, such as a PI3-Kinase inhibitor. In summary, our study provides a mechanistic insight and a rationale for Syk inhibition as a novel therapeutic option for the treatment of MM.

## Methods

### Cell culture

The cells lines AMO-1, U266, RPMI8226 and MM1-S were a kind gift from Helmut Salih from the University Hospital Tuebingen. The cells were cultured in RP10 medium (RPMI 1640 containing GlutaMAX, supplemented with 10% heat-inactivated fetal calf serum and 100 units/ml penicillin/streptomycin, all from Gibco, Karlsruhe, Germany) in a humidified atmosphere (37°C, 5% CO_2_). Cells were seeded into 75 cm^2^ flasks at 10^4^/10 ml/flask (BD Heidelberg, Deutschland).

After informed consent, blood samples were collected from patients with multiple myeloma hospitalized at the University Hospital Bonn. PBMCs were isolated by Ficoll/Paque (Biochrom, Berlin, Germany) density gradient centrifugation.

Cells were preincubated with zVAD (Bachem Distribution Services GmbH, Weil am Rhein, Germany) for 1 h. Piceatannol, applied at concentrations of 10, 25 and 50 µM (Sigma-Aldrich Chemie GmbH, Munich, Germany), R406, applied at concentrations of 1 and 5 µM, and BAY61-3606, applied at concentrations of 1, 2.5 and 5 µM, (Sigma-Aldrich Chemie GmbH, Munich, Germany) were added for 24 h. After 24 h, cells were prepared for further experiments.

To analyse possible synergistic effects we tested in addition thalidomide(MP Biochemicals, Solon, Ohio, USA), lenaldomide (Selleck Biochemicals, Houston, TX, USA) TRAIL (R&D Systems, Minneapolis, MN, USA), NVP-BEZ235 (Novartis Deutschland GmbH, Nürnberg, Germany), Bortezomib (Millenium Pharmaceuticals Limited, Cambridge, MA, USA) and five MAPK Inhibitors (PD98059, SB203580, SP600125, U0126, SB201290 all purchased from Tocris Bioscience, MO, USA).

### Preparation of whole cell lysates and nuclear extracts

For the generation of whole cell lysates, cells were lysed in RIPA buffer containing 1% Igepal CA-630, 0.5% Na-Deoxycholat, 0.1% SDS, 2 mM EDTA, 2 µg/ml Aprotinin, 1 mM PMSF. Protein concentration was measured using a bicinchoninic acid (BCA) assay (Pierce, Perbio Science, Bonn, Germany).

Nuclear extracts were prepared as described. In brief, 10^6^ cells were incubated in 400 µl Buffer A containing 10 mM HEPES pH 7.9, 10 mM KCL, 0.1 mM EDTA, 0.1 mM EGTA, 1 mM DTT and 0.5 mM PMSF. Cellular membranes were destroyed by addition of 10% Igepal CA-630 and vigorous vortexing. Nuclei were pelleted by centrifugation and resuspended in buffer C containing 20 mM HEPES pH 7.9, 0.4 M sodium chloride, 1 mM EDTA, 1 mM EGTA, 1 mM DTT and 0.5 mM PMSF. Nuclear proteins were recovered by centrifugation for 5 min at 20,000*g*.

### PAGE and Western blotting

20 µg of whole cell lysates or nuclear extracts of 1 × 10^6^ cells were separated by SDS-PAGE and subsequently transferred onto nitrocellulose membranes (Whatman, Dassel, Germany). The blots were probed with antibodies against bcl-x, cytochrome *c*, caspase-3, XIAP (all purchased from BD Biosciences, Heidelberg, Germany), ERK 1/2, mcl-1, survivin, Rel-A, Rel-B (all purchased from Santa Cruz Biotechnology, Inc., Santa Cruz, USA), p38, pp38 (all purchased from Cell Signaling Technology^®^, MA, USA). GAPDH (Santa Cruz Biotechnology, Inc., Santa Cruz, USA) was used as a loading control. Signals were detected using the ECL detection system (GE Healthcare, Munich, Germany).

### Immunoprecipitation

To estimate the expression of the protein tyrosine kinase syk in our multiple myeloma cell lines we performed immunoprecipitation. Cell lysates were assembled as explained above (see preparation of whole cell lysates). For immunoprecipitation Dynabeads^®^ Protein A or Protein G from Invitrogen (Life Technologies GmbH, Darmstadt, Germany) were used. The primary antibodies (3 µl/sample) were pooled to 50 µl Dynabeads^®^ Protein A and G. To isolate the generated complexes we washed (each with 200 µl AB Binding und Wasch Puffer) the solution and the suspension was passed by two magnets where the magnetic beads were reversibly trapped in the magnetic field and formed a compact dense plug.

To avoid co-elution of the antibody we cross linked the primary antibody to Dynabeads^®^. DSS was eluted in DMSO and a working solution consisting of 20× Coupling Buffer (2.5 µl), 2.5 mM DSS and ultrapure water (38.5 µl) was established. The Dynabeads^®^–antibody complex was resuspended in 50 µl working solution and incubated for 30–60 min in this solution.

Subsequent the Beads were resuspended with the whole cell lysates (100–500 µg protein). The Dynabeads^®^-antibody–antigen-complex was then incubated for 1 h, followed by a couple of steps of washing (3 × 200 µl Wash Buffer, 1× 100 µl Wash Buffer), magnetic field exposition and resuspending in 20 µl Elution buffer. Afterwards the pure precipitate was eluted from the beads of the supernatant and then analyzed by western blotting (explanation see PAGE and Western Blotting). The blots were probed with an antibody against p-syk (purchased from Santa Cruz Biotechnology, Inc., Santa Cruz, USA).

### Measurement of apoptosis

Apoptosis was measured by the method of Nicoletti [33]. 1 × 10^5^ cells were incubated in a hypotonic buffer containing 1% sodium citrate, 0.1% Triton X-100, 50 µg of propidium iodide per ml and subsequently measured by flow cytometry on a Cytomics FC 500 (Beckmann Coulter, Krefeld, Germany) using CXPAnalysis software. The hypodiploid cells to the left of the 2N peak were considered as apoptotic.

### Fluorimetric assay of caspase-3 activity

Caspase activity was determined from cytosolic extracts, by the cleavage of the fluorogenic caspase substrate *N*-acetyl-Asp-Glu-Val-Asp-aminomethylcoumarin (DEVD-AMC, Bachem, Heidelberg, Germany). In brief, for fluorimetric assay of caspase activity, cytosolic cell extracts were prepared by lysing cells in a buffer containing 0.5% NP-40, 20 mM HEPES pH 7.4, 84 mM KCl, 10 mM MgCl_2_, 0.2 mM EDTA, 0.2 mM EGTA, 1 mM DTT, 5 µg/ml aprotinin, 1 µg/ml leupeptin, 1 µg/ml pepstatin and 1 mM PMSF. Cell lysates were incubated with 50 mM DEVD-AMC in 200 µl buffer containing 37.5 mM HEPES pH 7.3, 75 mM NaCl, 7.5% sucrose, 0,075% CHAPS and 10 mM DTT. The release of aminomethylcoumarin was determined by fluorometry using an excitation length of 360 nm and an emission wave-length of 460 nm.

### Phenotyping of MM cells

Cells were treated as mentioned above. After incubation staining for fluorescence-activating cell sorting (FACS) followed. 5 × 10^5^ cells/tube were incubated with 10 µl anti-CD49d PE (purchased from BD Biosciences Europe, Erembodegem, Belgium), CD102 PE (purchased from BD Biosciences Europe, Erembodegem, Belgium) or CXCR4 PE (purchased from Beckman Coulter GmbH, Krefeld, Germany) and 10 µl anti-mouse IgG1, κ PE for 30 min at 18–22°C in the dark. When examined the next day, cells were fixed in 250 µl FACS-Buffer and 250 µl 2% formaldehyde and then analyzed by flow cytometry.

### Proliferation assay

Multiple myeloma cells were seeded into 96-well microtiter plates (Greiner Bio-One GmbH, Frickenhausen, Germany) at 10^6^, 10^5^ or 10^4^ cells/well and incubated for 24 h at 37°C, 5% CO_2_. The assay was performed in fourfold replicates. Cells were pulsed with ^3^H-thymidine (Hartmann Analytic, Braunschweig, Germany) and incubated for another 16 h at 37°C, 5% CO_2_. Cells were harvested then using a Filter Mate Harvester (Perkin Elmer, Waltham, USA) and uptake of ^3^H-thymidine was measured by a Microbeta TriLux (Perkin Elmer).

### Migration assay

Cells were seeded into transwell chambers (8 µm, BD) in 24-well plates (BD) and migration was induced through SDF-1α (Biovision, Mountain View, CA, USA).

After 3 h migrated multiple myeloma cells were analyzed by counting gated cells for a time interval of 60 s on a Cytomics FC 500 (Beckmann Coulter).

### TACS^®^-MTT cell proliferation assay

Measurement of cell viability and proliferation were performed with TACS^®^-MTT assay to quantify the cell population‘s response to Syk inhibitors. The yellow tetrazolium salt 3-4,5-dimethylthiazol-2yl]-2,5-diphenyl-tetrazolium bromide (MTT) is reduced by metabolic active cells and can then be quantitated by spectroscopic means. Measurement of proliferation and cell viability can be determined over the metabolic events that lead to apoptosis or necrosis.

Cells were cultured and treated with Syk Inhibitors as mentioned above and then plated in the wells. After adding the MTT agent, cells were incubated in the incubator until a purple dye was visible. In the next step Detergent Reagent was added and then the flat-bottomed 96-well plates were read after 2 h with the microplate reader at 570 nm. The average value was determined from triplicate reading.

### Syk inhibitors

Piceatannol (Sigma-Aldrich Chemie GmbH, Munich, Germany), a plant secondary product (3,4,3,5-tetrahydroxy-trans-stilbene), was identified as a tyrosine kinase inhibitor in thymocytes that also displays some activity against Lyn and STATs. The used concentrations were adjusted to reported working concentrations in literature. BAY61-3606 (Sigma-Aldrich Chemie GmbH, Munich, Germany), (orally available 2-(7-(3,4-dimethoxyphenyl)-imidazol(1,2-c)pyrimidin-5-ylamino)nicotinamide dihydrochloride), is a specific Syk inhibitor that acts as an ATP competitor blocking its catalytic activity and was shown to inhibit antigen-induced airway inflammation in rodents [34]. The used concentrations were adjusted to reported working concentrations in literature.

R406 is an adenosine triphosphate competitive, small molecule that was developed as a specific Syk inhibitor by Rigel Pharmaceuticals (San Francisco, CA, USA) with lower activity against FMS-like tyrosine kinase 3 (Flt3), JAK 1/2 and lymphocyte-specific protein tyrosine kinase (Lck) and is currently used in clinical trials for rheumatoid arthritis, idiopathic thrombocytopenic purpura (ITP), B-CLL and DLBCL. The used concentrations of this compound in our experiments represent serum levels obtained in treated patients. In volunteers and patients, plasma levels after a single dose of R406 between 1 and 5 µg/ml were reported [35].

### Statistics

Variance analysis was performed using SPSS 17.0 of SPSS Inc. Evaluation of synergistic effects was performed using SAS 9.2 from SAS Institute Inc.

## Additional file

**Additional file 1:** Effects of Syk-inhibitors are not due to cytotoxic effects. To quantify the cell population’s response to Syk inhibitors we performed measurement of cell viability and proliferation with TACS^®^-MTT. MM cell lines were incubated with Syk inhibitors (Piceatannol, R406 or Bay61-3606) and active cells were quantitated by spectroscopic means. Furthermore zVAD, a pan-caspase inhibitor, and triton 2%, as positive control, were added. Measurement of proliferation and cell viability within this MTT-assay can be determined over the metabolic events that lead to apoptosis or necrosis. The MTT assays showed no decline in cell viability when incubated with Syk inhibitors. The significance is related to DMSO/Ampuwa. ^+^P < 1.000, *P < 0.1, **P < 0.01, ***P < 0.001.
