# Supplementary figures and images for: Analysis of the anti-proliferative and the pro-apoptotic efficacy of Syk inhibition in multiple myeloma
Source: Exp Hematol Oncol. 2015 Aug 5;4:21. doi: 10.1186/s40164-015-0016-z (PMC4526421; doi:10.1186/s40164-015-0016-z)

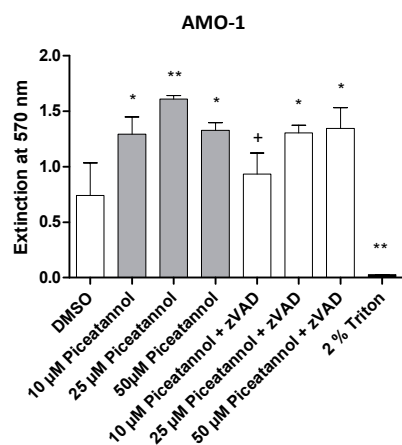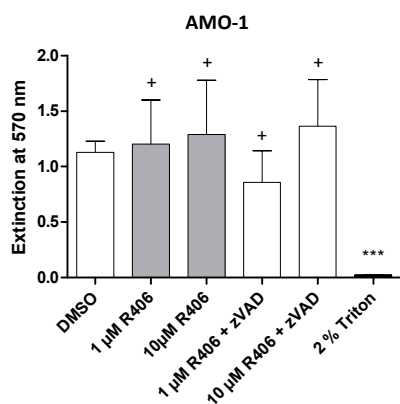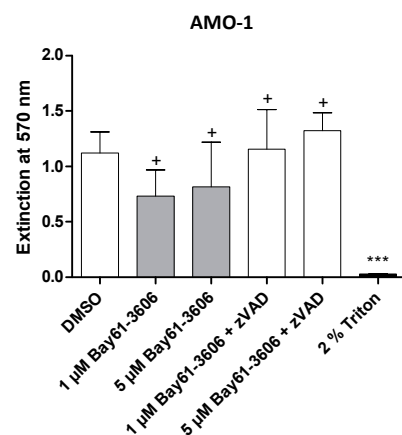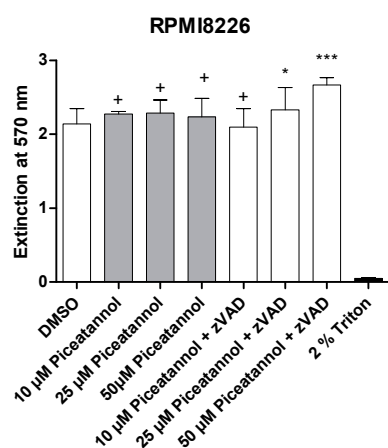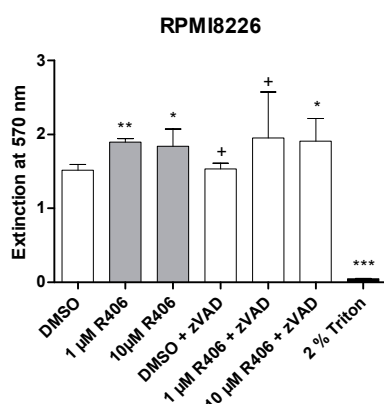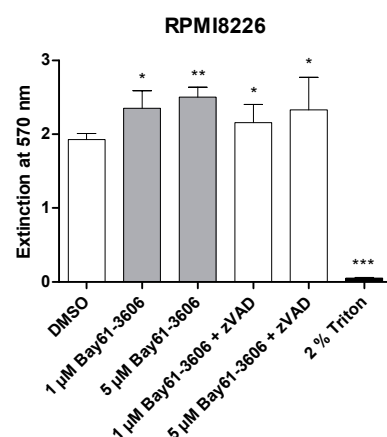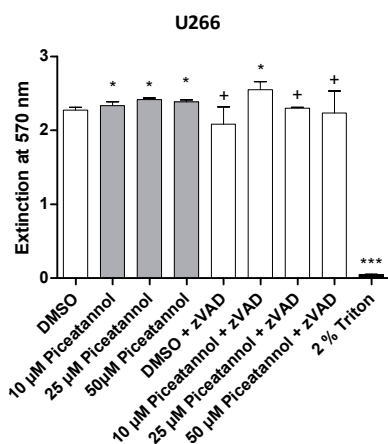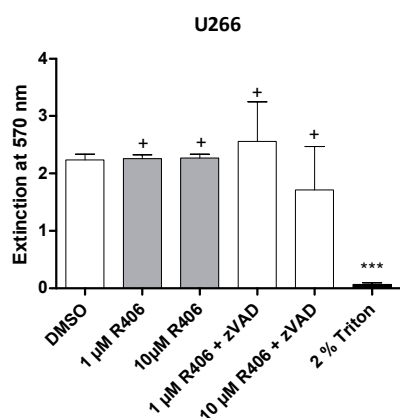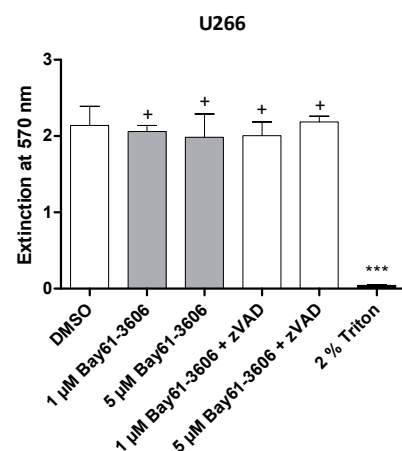

Supplement: Supplementary file 1 — Additional file 1: Effects of Syk-inhibitors are not due to cytotoxic effects. To quantify the cell population’s response to Syk inhibitors we performed measurement of cell viability and proliferation with TACS®-MTT. MM cell lines were incubated with Syk inhibitors (Piceatannol, R406 or Bay61-3606) and active cells were quantitated by spectroscopic means. Furthermore zVAD, a pan-caspase inhibitor, and triton 2%, as positive control, were added. Measurement of proliferation and cell viability within this MTT-assay can be determined over the metabolic events that lead to apoptosis or necrosis. The MTT assays showed no decline in cell viability when incubated with Syk inhibitors. The significance is related to DMSO/Ampuwa. +P < 1.000, *P < 0.1, **P < 0.01, ***P < 0.001. [file 40164_2015_16_MOESM1_ESM.pdf]
